# Real-World Experience on the Use of Eravacycline at Doses of 1 mg/kg Bodyweight and Fixed Dose Strategy in Two European Tertiary Centers

**DOI:** 10.3390/antibiotics15040421

**Published:** 2026-04-21

**Authors:** Karin Oberreiter, Miriam M. Moser, Lisa Schneider, Heinz Burgmann, Chiara Moreal, Simone Giuliano, Jacopo Angelini, Carlo Tascini, Matthias G. Vossen

**Affiliations:** 1 Clinical Division of Infectious Diseases and Tropical Medicine, Department of Medicine I, Medical University of Vienna, 1090 Vienna, Austria; 2 Department of Neurosurgery, Medical University of Vienna, 1090 Vienna, Austria; 3 Department of Medicine (DMED), University of Udine, 33100 Udine, Italy; 4 Infectious Diseases Clinic, Department of Specialistic Medicine, University Hospital of Central Friuli, 33100 Udine, Italy; 5 Clinical Pharmacology and Toxicology Institute, University Hospital Friuli Centrale ASUFC, 33100 Udine, Italy

**Keywords:** eravacycline, dose strategy, multidrug-resistant

## Abstract

**Background:** Eravaycline is a novel fully synthetic fluorocycline that is currently approved for complicated intra-abdominal infections. However, it is sometimes also used off-label in tertiary care centers for other infection sites as an antibiotic of last resort due to its broad spectrum of activity and efficacy against *Enterobacterales*, including multidrug-resistant pathogens like extended spectrum β-lactamase (ESBL) producers or carbapenem-resistant *Enterobacterales*, as well as all Gram-positive organisms including methicillin-resistant *Staphylococcus aureus* (MRSA) and vancomycin- and linezolid-resistant *Enterococcus faecium* (VRE). **Methods:** We retrospectively included a total of 78 patients from Austria and Udine who received eravacycline between April 2023 and August 2024 to evaluate the real-world efficacy of eravacycline in various infection sites and pathogens using descriptive statistics. **Results:** Eravacycline was most commonly used in intra-abdominal infections (44.9%), followed by pneumonia (12.8%) and infections of unknown origin (7.7%)*. Escherichia coli*, including ESBL producers, was the most common pathogen (24.4%), followed by *Enterococcus* spp. (12.8%) and *Klebsiella pneumoniae* (12.8%). Clinical cure was achieved in 65% of patients, whereas microbiological cure was documented in 46%; source control was attained in 48.7%, and 16.7% died within 30 days. A total of 48% of patients required intensive care. **Conclusions:** Eravacycline represents a possible therapeutic option for a wide range of pathogens, but its use must be evaluated in the context of infection site and severity.

## 1. Introduction

Eravacycline is a novel fully synthetic fluorocycline that was developed to overcome the resistance mechanisms of tetracyclines. A change in the C-7 and C-9 position of the tetracycline nucleus has been shown to increase the antibacterial potency, whilst the mechanism of action remains similar to other members of the tetracycline class, as it also inhibits protein synthesis by binding to the ribosome [1,2,3]. A change in the C-9 position has been demonstrated to significantly reduce the affinity of the efflux pumps of the tet(A), tet(B) and tet(K) class against the molecule, whereby the activity against *Staphylococci*, *Klebsiella pneumoniae* and *Escherichia coli* in particular could be increased [3,4,5]. Resistance to eravacycline may be conferred only by tet(X) tetracyclinases [6].

Eravacycline is currently authorized for the treatment of complicated intra-abdominal infections [7]. Austria is one of the first countries in Europe where eravacycline is widely used. Whilst it is currently authorized for the treatment of complicated intra-abdominal infections, it may also be beneficial in other scenarios, particularly in cases of infections by extended-spectrum β-lactamase-producing or carbapenem-resistant *Enterobacterales* [8,9,10]. Antibiotics of last resort, such as eravacycline, are frequently used off-label in the context of tertiary care centers.

The objective of this retrospective study was to analyze real-world data and evaluate the efficacy of the on- and off-label use of eravacycline at two tertiary care centers (Medical University of Vienna and the University of Udine).

## 2. Results

A total of 78 patients who received eravacycline between April 2023 and August 2024 at the Medical University of Vienna and the University of Udine were analyzed in this study. Of these patients, 28 (35.9%) were female and 50 (64.1%) were male. The mean age of the patients was 60 ± 17 years. A total of 38 patients (48.7%) received eravacycline in an intensive care setting, while 39 patients (50%) were treated on a general ward. One patient received eravacycline as outpatient parenteral antimicrobial treatment at an outpatient clinic (Table 1).

### 2.1. Pathogens and Infection Sites

**Figure 1:**
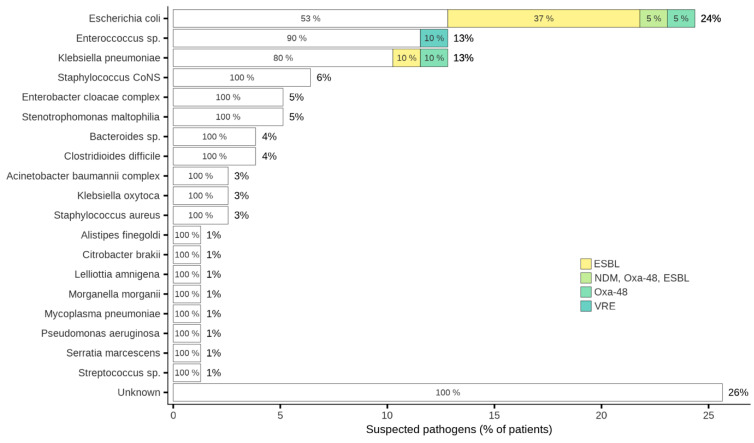
Pathogens identified, including resistance mechanisms.

A broad spectrum of suspected pathogens was cultivated from samples in the patients who received eravacycline (Table 2, Figure 1). The most prevalent pathogen was *Escherichia coli* (19 patients, 24.4%), including various resistance mechanisms (ESBL, NDM, Oxa-48, details in Table 2), followed by *Enterococcus* sp. (10 patients, 12.8%, with one *Enterococcus faecium* VRE) and *Klebsiella pneumoniae* (10 patients, 12.8%) with different resistance mechanisms (ESBL, Oxa-48). *Coagulase negative Staphylococci* were identified in five patients (6.4%) (Table 2), while *Staphylococcus aureus* was detected in two patients (2.6%), one of which was methicillin-resistant *staphylococcus aureus* (MRSA). *Bacteroides* spp. was identified in three patients (3.8%), *Pseudomonas aeruginosa* strain classified as 3-MRGN was isolated from one patient (1.3%), *Stenotrophomonas maltophilia* was detected in four patients (5.1%), and *Clostridioides difficile* was present in three patients (3.9%). The prevalence of other pathogens was 2.6% or less, listed in greater detail in Table 2.

The decision to initiate eravacycline therapy was precipitated by a range of infectious foci. A total of 35 patients (45%) with complicated intra-abdominal infections were treated with eravacycline in accordance with its current approval. Ten patients (13%) suffered from severe pneumonia. In six cases (8%) the infectious focus remained unidentified. Skin and soft tissue infection, *Clostridioides difficile*-associated diarrhea, burn-related acute bacterial skin and soft-tissue infection, and urosepsis each represented the indication for eravacycline treatment in three patients (3.9%). In two cases (2.6%), the initiation of eravacycline therapy was due to sepsis with unknown origin. In addition, each of the following conditions represented the indication for eravacycline therapy in one individual patient (1.3%): bloodstream infection, central line-associated bloodstream infection, bullous erysipelas, complicated urinary tract infection, epididymiditis, orchitis, fasciitis, open fracture-associated infection, endocarditis, constrictive pericarditis, mediastinitis, pyelonephritis, and spondylodiscitis. In the present study, eravacycline was administered to a total of 24 patients (30.8%) with a positive blood culture. Of these, seven *Escherichia coli* isolates were found, including three ESBL-producing strains, and one carbapenemase producer additionally harboring both NDM and OXA-48. Furthermore, five *Klebsiella pneumoniae* isolates were detected, one of which was classified as 4-MRGN. Four *Enterococcus* spp. were detected in blood cultures, two of which were classified as vancomycin-resistant enterococci (VRE). *Stenotrophomonas maltophilia* was identified on two occasions in blood cultures, and one case of *Acinetobacter baumannii* complex classified as 4-MRGN was also identified in blood culture. Follow-up blood cultures were obtained in 23 patients; four demonstrated persistent bacteremia.

### 2.2. Therapy

At the Medical University of Vienna, the eravacycline dose was calculated at 1 mg/kg in 6% of cases. In 84% of cases, patients received a fixed dose regimen of eravacycline corresponding to the next highest multiple of 50 mg. In Udine, every patient received a dose calculated at 1 mg/kg. In general, the total daily dose was found to be lower in the Udine group than in the Medical University of Vienna. A total of twenty-two patients received eravacycline in a single daily dose, 55 patients received it twice daily, and one patient received three doses of the drug over the course of a day. Nineteen patients received a total daily dose of 200 mg or 300 mg. The most frequently administered regimen was 100 mg twice per day (Table 3). On average, patients with a positive blood culture received 180 mg of eravacycline per day, while patients with any other focus of infection received a median of 200 mg eravacycline per day.

Among those 19 patients who received daily doses of 200 or 300 mg of eravacycline, two patients died—one after two days and one after six days from therapy initiation. In both patients, source control was not achieved, and microbiological cure was not achieved. One patient had an extensive malignant disease, while the other patient underwent aortic valve replacement due to endocarditis caused by *Candida* spp. The latter patient was prescribed eravacycline following cefiderocol treatment failure in a 4-MRGN *Klebsiella pneumoniae* catheter-related bloodstream infection. In the fixed dose cohort, 14 of the 19 (73.6%) patients achieved clinical cure, compared with 65% in the overall population. All patients in this fixed dose cohort did not have documented side effects.

Nine patients (11.5%) received eravacycline as monotherapy, and 30 patients (38.5%) received a pseudomonas-effective combination therapy. In 13 patients (16.7%), the presence of combination therapy could not be ascertained due to missing documentation.

**Figure 2:**
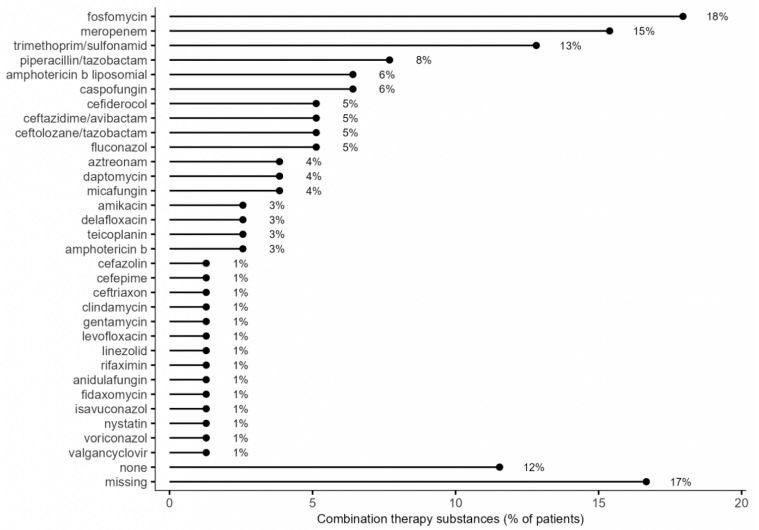
Frequencies of drugs used in combination with eravacycline.

A total of 29 patients (37.2%) received a combination of two antimicrobial agents including eravacycline, 18 patients (23.1%) received three agents, six patients (7.7%) received four agents, one patient (1.3%) received five agents, and two patients (2.6%) received a total of seven agents including eravacycline (details of the respective combinations are provided in Table 4, Figure 2).The most common combination partner for eravacycline was fosfomycin, which was the case in 14 patients (18%), followed by meropenem in 12 patients (15.4%) and trimethoprim/sulfamethoxazole in 10 patients (13%), but only six of those received a therapeutic dose (additional details are provided in Table 4, Figure 2).

**Figure 3:**
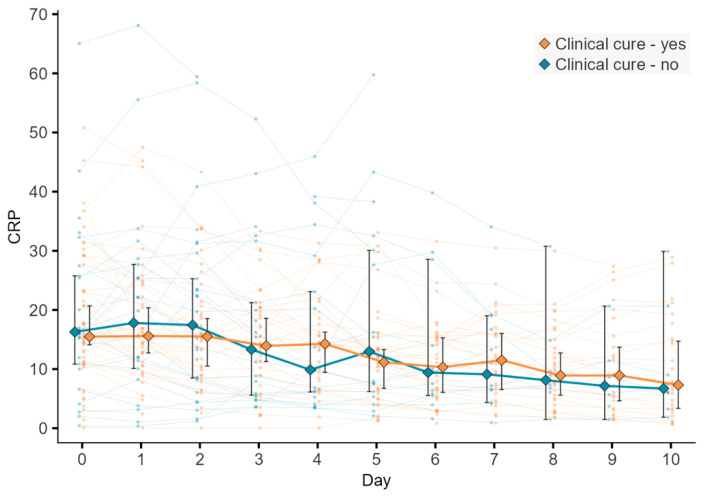
C-reactive protein level over the course of eravacycline therapy stratified by clinical cure vs. no clinical cure.

At the initiation of eravacycline therapy, the median C-reactive protein (CRP) level was 15.9 mg/dL (interquartile range (IQR) 14.6). After five days, the CRP had decreased to 12 mg/dL (IQR 10.2), corresponding to a median reduction of −6.7 mg/dL (IQR 13.2). After ten days, the median CRP further declined to 7.1 mg/dL (IQR 11.7), corresponding to a median reduction of −9.3 mg/dL (IQR 9.7) compared with baseline levels. Concomitantly, the median white blood cell count was 12.3 G/L (IQR 9.4) at treatment initiation, increasing to 14.5 G/L (IQR 11.9) after five days (median change +0.9 G/L, IQR 1.9) and subsequently decreasing to 10.3 G/L (IQR 9.7) after ten days, reflecting a median reduction of −5.3 G/L (IQR 12.8). Differences in CRP levels between clinical cure vs. no clinical cure are shown in Figure 3.

There were very few documented side effects. In three patients, liver enzyme elevation occurred during eravacycline therapy, another patient developed thrombocytopenia, and in a further patient hyperbilirubinemia led to discontinuation of eravacycline therapy.

### 2.3. Outcome

Clinical cure was achieved within 28 days in 51 patients (65%; CI 0.55–0.76), with a documented improvement in the general condition. Microbiological cure was obtained in 36 patients (46%; CI 0.35–0.57) and adequate source control was achieved in 38 patients (49%; CI 0.38–0.6). Multivariate analysis demonstrated that achievement of source control was more strongly associated with outcome than other parameters (*p* < 0.001) (Table 5). However, this multivariate analysis should be interpreted with caution, as the analysis is exploratory and the study was not powered to address this specific research question. A total of 25 patients (32%; CI 0.2–0.42) experienced a breakthrough infection, and 13 patients (16.7%) died within 28 days after initiation of eravacycline treatment (Table 1). Among the 24 patients with positive blood cultures, 17 patients achieved both clinical and microbiological cure, while five of these patients died (Table 6). Patients with a positive blood culture for *Klebsiella pneumoniae*, *Enterococcus* spp. and *Stenotrophomonas maltophilia* generally survived. Of the five deceased patients with isolated blood stream infections, the identified pathogens were *E. coli*, *Bacteroides fragilis* and *Enterobacter cloacae*.

Follow-up blood cultures were available in 23 patients: 19 had negative results in the follow-up blood culture, whereas four patients remained positive in the repeated blood culture under therapy.

No significant differences in outcome frequencies were found between eravacycline monotherapy and combination therapies using Fisher’s exact test (*p* ≥ 0.153) (Table 7). The proportion of clinical cure and source control was significantly higher in the patient subgroups treated in the general ward or at the outpatient clinic, likely reflecting the less severe infections (Table 6).

## 3. Discussion

In this study including 78 patients who received eravacycline in a real-world setting in Austria (Vienna) and Italy (Udine), clinical cure was achieved in 65% of cases, microbiological cure in 46%, and source control in 49%. The primary indication was intra-abdominal infection. The most frequently identified pathogens were *Escherichia coli* featuring various resistance mechanisms, followed by *Enterococcus* spp. and *Klebsiella pneumoniae*. A similar real-world study has previously been published for the US population [11], but as resistance patterns in Europe differ substantially from those in North America, investigation of eravacycline use in European patients is essential. Eravacycline has been approved for the treatment of intra-abdominal infections [7]. The high volume of distribution may explain its efficacy in treating abdominal infections [12].

However, in the present study it was also frequently used for the management of pneumonia and infections of unknown origin. Prior analyses have suggested the possible efficacy of eravacycline in pneumonia [13]. However, the IGNITE phase 2 study reported treatment failure in lobar pneumonia [14]. This is in line with our study, where more than half of the pneumonia patients did not achieve clinical cure. In contrast to IGNITE, not all pneumonia cases in our cohort had pathogen identification, and eravacycline was often initiated as a last-line therapy in severely ill patients. This likely contributed to the finding of less than 50% clinical cure in this subpopulation. Although eravacycline has demonstrated favorable pharmacokinetics for pulmonary infections, similarly to our findings, Scott et al. reported poor outcomes among pneumonia patients in their retrospective analysis, in which all non-survivors also had bacteremia [15,16]. In another real-world study of patients with positive sputum cultures for *Enterococcus* spp., *K. pneumoniae*, or *Stenotrophomonas maltophilia*, survival was also poor [11]. Nevertheless, studies in healthy volunteers have demonstrated excellent pulmonary penetration, achieving up to 50-fold higher concentrations in alveolar macrophages than in plasma [12,17]. These findings suggest that eravacycline may still be effective in treating pulmonary infections. Overall, there is still a lack of evidence for the use of eravacycline in the treatment of pneumonia and bacteremia, and further prospective studies are required.

Eravacycline is presumed to have reduced effectiveness in bloodstream infections due to its high protein binding, which lowers free plasma concentrations [12,18]. In addition, as a tetracycline derivative, it is primarily bacteriostatic, which may be inadequate for achieving rapid blood sterilization. Notwithstanding these limitations of eravacycline in bloodstream infections, only five patients with positive blood cultures in our cohort died within 30 days, and follow-up cultures were negative in 23 patients. This outcome may be related to the relatively high median eravacycline dose of 200 mg/day used at our centers, which exceeds current treatment recommendations [19].

The clinical cure rate for fixed doses of eravacycline was found to be higher (73.6%) than in the overall population (65%). Two of the 19 patients who received fixed daily doses of 200 or 300 mg eravacycline died. However, both were treated with severe underlying conditions, including an advanced malignant disease and endocarditis following aortic valve replacement. Consequently, given the small cohort size and the retrospective study design, no definitive conclusions can be drawn regarding fixed dose therapy recommendations.

As a new fully synthetic fluorocycline, eravacycline is active against Gram-negative pathogens, such as *Escherichia coli* and *Klebsiella pneumoniae*, as well as Gram-positive pathogens including *Enterococcus* spp. and *Staphylococci* [20]. These were also the most common initial pathogens in the approval studies, where eravacycline and ertapenem showed comparable efficacy [14]. Kunz Coyne et al. reported predominant use of eravacycline in infections caused by *Enterobacterales* (including carbapenem-resistant strains), *Acinetobacter* spp. (including carbapenem-resistant *Acinetobacter* spp.), *Enterococci* spp. (including vancomycin-resistant *Enterococci* spp.), and *Stenotrophomonas maltophilia* [11]. In contrast, *Stenotrophomonas maltophilia* and *Acinetobacter* spp., including 4-MRGN isolates, were rarely observed in our study, underscoring regional differences in pathogen distribution between Europe and North America.

A total of 48.7% of patients who received eravacycline in our centers required intensive care, a proportion higher than that reported in US real-world data (42.5%) [11] and substantially higher than in the cohort described by Van Hise et al., where only 2% of patients required long-term acute care, with Intensive Care Unit (ICU) status not reported [21]. Due to the severity of illness and frequent detection of multiple pathogens with resistance mechanisms, combination therapy was commonly used in our cohort, most often using fosfomycin, meropenem and trimethoprim/sulfamethoxazole, while only 11.5% received eravacycline as monotherapy. A multicenter retrospective US study found similar results in terms of outcome, where *Klebsiella pneumoniae* was the most frequently detected pathogen [22]. In the IGNITE 4 study, the clinical cure rate ranged from 90.8% to 96.9% among patients with intra-abdominal infections—patients with a predicted survival of less than 6–8 weeks were excluded [10]. Van Hise et al. reported a clinical cure rate of 94% in patients treated with eravacycline; however, the study included only patients treated in general wards or by primary care physicians [21]. In comparison, our outcomes were less favorable: clinical cure was achieved in only 65% of cases within 30 days. The large number of patients requiring intensive care, in conjunction with the frequent use of eravacycline as part of combination regimens, suggests that eravacycline was mostly used as a last-line agent in the present study. In this study, survival rate was 83.3%, which is approximately 10% lower than in North America (USA and Canada) where real-world studies have reported survival rates of 94% [21] and 94.7% [11]. In the aforementioned studies, non-survivors commonly had positive sputum or blood cultures for *Enterococcus* spp., *Klebsiella pneumoniae*, or *Stenotrophomonas maltophilia* [11]. In our study, these pathogens were also among the most frequently identified organisms. As demonstrated in the IGNITE 4 study, these pathogens were frequently isolated in patients with complicated intra-abdominal infections. Furthermore, eravacycline was proven to be non-inferior to meropenem, with a clinical response rate of approximately 90% in both arms of the IGNITE 4 trial [10]. Further prospective studies focusing on these pathogens in non-abdominal infections are required to evaluate the efficacy of eravacycline for other indications to identify factors contributing to poor outcomes.

Given its activity against multidrug-resistant pathogens and its higher antibacterial activity compared to tigecycline and ertapenem, eravacycline might be a promising option for treating *Clostridioides difficile* infections [23,24]. The ESCMID guidelines [25] already recommend tigecycline for *Clostridioides difficile* infections. Eravacycline, as a tigecycline derivative, may have the potential to be equally effective, but there is currently a lack of evidence to support this. Tsai et al. demonstrated its in vitro activity against *Clostridioides difficile* [24]. A small proportion of *Clostridioides difficile* infections in our study (3.9%) and in the cohort of Kunz Coyne et al. (6.5%) were treated with eravacycline, and all patients in our cohort achieved clinical cure [11].

Median CRP levels demonstrated a downward trend within a period of 10 days, while the median leukocyte count fluctuated. Importantly, these inflammatory parameters may have been influenced by several confounding factors. For instance, many ICU patients underwent surgical interventions during eravacycline therapy, which could have independently affected leukocyte and CRP levels. Moreover, severe illness and systemic inflammation may alter the pharmacokinetics of eravacycline and concomitant antibiotics, thereby potentially impacting therapeutic response [26]. Consequently, these findings should be interpreted with caution, and prospective studies including pharmacokinetic monitoring are warranted.

Assessment of clinical adverse effects was limited due to the retrospective design of the study. Only a few treatment-emergent adverse events (TEAEs) were documented in our cohort. Three patients had an increase in liver function tests, one patient developed thrombocytopenia, and in one patient an increase in bilirubin lead to the discontinuation of the therapy. The frequency of TEAEs is consistent with the phase 2 IGNITE study, where approximately one quarter of patients reported mild, mostly gastrointestinal, side effects [14].

## 4. Limitations

Due to the retrospective nature of our trial, important confounding factors might not have been captured. The use of eravacycline as a last-line therapy in our centers and the decision to include all patients that received eravacycline during the inclusion period may negatively bias the reported treatment outcome. We deliberately chose this approach as we deemed a positive bias more dangerous. Due to the absence of systematic documentation in this retrospective study, patient body weight could not be consistently ascertained. Consequently, eravacycline dosing regimens were not reliably traceable, representing a significant limitation of the study. Moreover, MIC data were not available for the majority of included patients, precluding their analysis in this study. Another limitation is the relatively small sample size which restricts the robustness of subgroup analyses, particularly regarding treatment effects at different infection sites. Analysis of the effect on different infection sites needs to be further analyzed in prospective studies. Another limiting factor was that the outcome parameter clinical cure, defined as no further hospital admissions, could not be fully verified, as readmissions to external hospitals were not recorded. Finally, adverse events may have been insufficiently documented and therefore potentially underreported in some patients.

## 5. Material and Methods

In this multicenter exploratory cohort study, a retrospective analysis was conducted on 78 patients (63 from Vienna and 15 from Udine) who received eravacycline due to an infection between April 2023 and August 2024. In both centers, susceptibility was assessed using EUCAST guidelines.

Patients were included in the study if they had been admitted to either the Medical University of Vienna or the University of Udine Medical Center and had been treated with eravacycline for a minimum of 72 h. Screening was performed through pharmacy dispensation logs. As a result, this study represents a cross-section of patients in which eravacycline was deemed the adequate therapeutic option by two independent infectious disease specialists (dispensation of the drug requires authorization from a senior infectious disease physician, and prescription was performed by infectious disease physicians only). Patients below the age of 18 years were excluded from the study. In total, five patients were excluded from the study due to a lack of documentation. An analysis was conducted on the treatment, with the following parameters being considered: indication, bacterial spectrum and resistance, dosage per day and number of doses per day, mortality, as well as clinical and microbiological cure. The clinical cure (% clinical cure) was defined as the assessment of treatment success by two infectious disease specialists, who evaluated treatment success based on the following parameters: fever, the course of inflammatory parameters, the level of catecholamines consumed, the patient’s overall condition (as documented by the treatment team), and the necessity of subsequent hospitalization following discharge. The microbiological cure (% microbiological cure) was determined by the absence of subsequent positive follow-up cultures with the initial pathogen up to 30 days following the end of eravacycline therapy. The primary outcome measure was clinical cure. The secondary outcome measure was the proportion of patients with a microbiological cure. In addition, a comprehensive analysis was conducted to ascertain the types of pathogens treated, the combination therapy partner, the source of infection, and the inflammatory parameters (CRP, PCT, IL-6, white blood cell count). Furthermore, the duration of therapy, 30-day mortality, length of hospitalization, source control, breakthrough infections, and eravacycline-associated side effects were analyzed.

### Statistical Analysis

Categorical and nominal variables (e.g., gender) are presented as absolute frequencies and relative frequencies (%). Numerical variables (e.g., age) are presented as median with interquartile range. The distribution of numerical data, i.e., inflammatory parameters, is visualized in dot graphs. All calculations and graphical presentation were performed using R (Version 4.5.2, R Foundation for Statistical Computing, Vienna, Austria, https://www.R-project.org/).

## 6. Conclusions

In our multicenter retrospective study of 78 patients treated with eravacycline, clinical cure within 28 days was achieved in 65% of cases and microbiological cure in 46%. Nevertheless, 32% of patients experienced breakthrough infections and overall mortality remained high, reflecting the severity of infections in this cohort. Importantly, the second and third most frequent indications for treatment were infections of unknown origin and pneumonia. Our findings indicate that eravacycline can achieve favorable clinical and microbiological responses in pretreated patients; however, its effectiveness in critically ill patients, particularly across different sites of infection, warrants further prospective studies.

## Figures and Tables

**Table 1:** Demographics.

Patient Characteristics	
Treatment center	*n* [%]
University of Udine	15 [19.2]
Medical University of Vienna	63 [80.8]
Treatment Unit	*n* [%]
Outpatient clinic	1 [1.3]
Intensive care unit	38 [48.7]
Ward	39 [50]
Gender	*n* [%]
Male	50 [64.1]
Female	28 [35.9]
Age	Mean [SD]
	59.35 [17.4]
Outcome	
	*n* [%]
Clinical cure	51 [65.4]
Microbiological cure	36 [46.2]
Source control	38 [48.7]
Death	13 [16.7]

**Table 2:** Responsible pathogens for treatment start with eravacycline. MRGN = multi-resistant Gram-negative bacteria, ESBL = extended-spectrum beta-lactamase, OXA48 = oxacillinase-48, sp. = species.

Identified Pathogens	*n*	[%]
*Escherichia coli*	19	24.3
ESBL	7/19	
NDM, Oxa-48, ESBL	1/19	
Oxa-48	1/19	
MRGN (not otherwise specified)	1/19	
*Enteroccoccus* spp.	10	12.8
VRE	1/10	
*Klebsiella pneumoniae*	10	12.8
ESBL	1/10	
4 MRGN	1/10	
OXA48	1/10	
*Staphylococcus coagulase* negative	5	6.4
*Enterobacter cloacae* complex	4	5.1
*Stenotrophomonas maltophilia*	4	5.1
*Bacteroides* spp.	3	3.9
*Pseudomonas aeruginosa*	1	1.3
3-MRGN	1/1	
*Staphylococcus aureus*	1	1.3
MRSA	1/1	
*Clostridioides difficile*	3	3.9
*Acinetobacter baumannii* complex	2	2.6
4-MRGN	2/2	
*Citrobacter koseri*	1	1.3
*Klebsiella oxytoca*	2	2.6
VIM	1/2	
*Morganella morganii*	1	1.3
*Serratia marcescens*	1	1.3
*Streptococcus* sp.	1	1.3
*Alistipes finegoldi*	1	1.3
*Citrobacter brakii*	1	1.3
*Lelliottia amnigena*	1	1.3
*Mycoplasma pneumoniae*	1	1.3
Unknown	20	25.6

**Table 3:** Doses and administration intervals of eravacycline.

Total Dose Per Day (mg)	*n* (%)	*n* (%) Vienna	*n* (%) Udine
100	8 (10.26)	6 (9.52)	2 (13.33)
120	2 (2.56)	1 (1.59)	1 (6.67)
130	1 (1.28)	-	1 (6.67)
140	3 (3.85)	1 (1.59)	2 (13.33)
150	7 (8.97)	3 (4.76)	4 (26.67)
160	3 (3.85)	1 (1.59)	2 (13.33)
170	1 (1.28)	1 (1.59)	-
180	3 (3.85)	-	3 (20)
200	44 (56.41)	44 (69.84)	-
240	1 (1.28)	1 (1.59)	-
300	5 (6.41)	5 (7.94)	-
Dose per administration	*n* (%)	*n* (%) Vienna	*n* (%) Udine
100	30 (38.46)	30 (47.62)	-
120	1 (1.28)	1 (1.59)	-
150	3 (3.85)	3 (4.76)	-
200	16 (20.51)	16 (25.4)	-
300	3 (3.85)	3 (4.76)	-
50	7 (8.97)	5 (7.94)	2 (13.33)
60	2 (2.56)	1 (1.59)	1 (6.67)
65	1 (1.28)	-	1 (6.67)
70	3 (3.85)	1 (1.59)	2 (13.33)
75	5 (6.41)	1 (1.59)	4 (26.67)
80	3 (3.85)	1 (1.59)	2 (13.33)
85	1 (1.28)	1 (1.59)	-
90	3 (3.85)	-	3 (20)

**Table 4:** Concomitant antimicrobial and antifungal therapy with eravacycline.

Combination Therapy	*n* (%)
**One combination partner**	**29 (37.18)**
PiperacillinTazobactam	5 (6.41)
Fosfomycin	4 (5.13)
Trimethoprim/Sulfonamid	3 (3.84)
Cefiderocol	2 (2.56)
Ceftazidime/Avibactam	2 (2.56)
Ceftolozane/Tazobactam	2 (2.56)
Daptomycin	2 (2.56)
Meropenem	2 (2.56)
Aztreonam	1 (1.28)
Caspofungin	1 (1.28)
Cefepime	1 (1.28)
Ceftriaxon	1 (1.28)
Delafloxacin	1 (1.28)
Fluconazol	1 (1.28)
Teicoplanin	1 (1.28)
**Two combination partners**	**18 (23.08)**
Amphotericin b liposomial and Meropenem	2 (2.56)
Fosfomycin and Meropenem	2 (2.56)
Fosfomycin and Trimethoprim/Sulfonamid	2 (2.56)
Amphotericin b liposomial and Cefiderocol	1 (1.28)
Amphotericin b liposomial and Ceftolozane/Tazobactam	1 (1.28)
Anidulafungin and Cefiderocol	1 (1.28)
Caspofungin and Ceftazidime/Avibactam	1 (1.28)
Caspofungin and Linezolid	1 (1.28)
Caspofungin and Trimethoprim/Sulfonamid	1 (1.28)
Clindamycin and Fosfomycin	1 (1.28)
Fluconazol and Fosfomycin	1 (1.28)
Fosfomycin and Voriconazole	1 (1.28)
Levofloxacin and Piperacillin/Tazobactam	1 (1.28)
Meropenem and Teicoplanin	1 (1.28)
Meropenem and Trimethoprim/Sulfonamid	1 (1.28)
**Three combination partners**	**6 (7.69)**
Amikacin, Amphotericin b and Nystatin	1 (1.28)
Amphotericin b liposomial, Ceftolozane/Tazobactam and Meropenem	1 (1.28)
Aztreonam, Fidaxomycin and Micafungin	1 (1.28)
Aztreonam, Micafungin and Trimethoprim/Sulfonamid	1 (1.28)
Fluconazol, Fosfomycin and Micafungin	1 (1.28)
Daptomycin, Delafloxacin and Meropenem	1 (1.28)
**Four combination partners**	**1 (1.28)**
Fosfomycin, Isavuconazol, Trimethoprim/Sulfonamid and Valgancyclovir	1 (1.28)
**Six combination partners**	**2 (2.56)**
Amikacin, Cefazolin, Fluconazol, Fosfomycin, Gentamycin and Meropenem	1 (1.28)
Amphotericin b, Caspofungin, Ceftazidime/Avibactam, Meropenem, Rifaximin and Trimethoprim/Sulfonamid	1 (1.28)

**Table 5:** Multivariate analysis on potential confounders.

	Clinical Cure		Microbiological Cure		Death	
Variable	OR (95% CI)	*p*	OR (95% CI)	*p*	OR (95% CI)	*p*
Intensive care unit	0.82 (0.66–1.03)	0.088	1.13 (0.85–1.49)	0.4	1 (0.8–1.25)	0.996
Source control	1.7 (1.38–2.08)	<0.001	1.79 (1.38–2.31)	<0.001	0.82 (0.66–1.01)	0.064
Eravacycline Monotherapy	0.92 (0.69–1.23)	0.585	1.25 (0.89–1.77)	0.208	0.99 (0.74–1.32)	0.931
Abdominal infection	1.11 (0.92–1.34)	0.293	0.91 (0.72–1.14)	0.41	0.97 (0.8–1.18)	0.779
Positive blood culture	1.02 (0.83–1.24)	0.884	1.37 (1.08–1.74)	0.013	1.08 (0.88–1.33)	0.476

**Table 6:** Clinical and microbiological outcomes—differences between admission sites and infection sites.

Outcome, *n* (%)	All Patients (*n* = 78)	ICU (*n* = 38)	Ward/Outpatient (*n* = 40)	*p*	Intra-Abdominal (*n* = 35)	Off-Label (*n* = 43)	*p*	Positive Blood Culture (*n* = 24)	No Positive Blood Culture (*n* = 54)	*p*	Source Control (*n* = 38)	No Source Control (*n* = 38)	*p*
Clinical cure	51 (65.4)	18 (47.4)	33 (82.5)	0.002	24 (68.6)	27 (62.8)	0.768	17 (70.8)	34(63)	0.610	36 (94.7)	14 (36.8)	<0.001
Microbiological cure	36 (46.2)	16 (42.1)	20 (50)	0.159	15 (42.9)	21 (48.8)	0.905	18 (75)	18 (33.3)	0.150	25 (65.8)	11 (28.9)	<0.001
Source control	38 (48.7)	12 (31.6)	26 (65)	0.006	17 (48.6)	21 (48.8)	1.000	12 (50)	26 (48.1)	1.000	-	-	-
Death	13 (16.7)	8 (21.1)	5 (12.5)	0.478	6 (17.1)	7 (16.3)	1.000	5 (20.8)	8 (14.8)	0.524	3 (7.9)	10 (26.3)	0.068

**Table 7:** Outcomes across different eravacycline treatment strategies (monotherapy and combination therapies).

Outcome, *n* (%)	Eravacycline Monotherapy (*n* = 9)	Any Kind of Combination (*n* = 56)	*p*-Value	1 Combination Partner (*n* = 29)	2 Combination Partners (*n* = 18)	≥3 Combination Partners (*n* = 9)
Clinical cure	7 (77.8)	35 (62.5)	0.47	19 (65.5)	13 (72.2)	3 (33.3)
Microbiological cure	6 (66.7)	25 (44.6)	0.24	11 (37.9)	10 (55.6)	4 (44.4)
Source control	6 (66.7)	24 (42.9)	0.29	11 (37.9)	10 (55.6)	3 (33.3)
Death	1 (11.1)	10 (17.9)	1.00	7 (24.1)	2 (11.1)	1 (11.1)

## Data Availability

Dataset available on request from the authors due to containing information that could compromise the privacy of research participants.
